# Differential Expression of Blood Group Precursor Antigen in Human Breast Cancer Tissue

**DOI:** 10.31487/j.ijcst.2020.01.04

**Published:** 2020-08-14

**Authors:** Yi Jiang, Denong Wang

**Affiliations:** Tumor Glycomics Laboratory, SRI International Biosciences Division, Menlo Park, California, USA

**Keywords:** Blood group precursors, breast cancer, circulating tumor cells, cancer stem cells, glycan marker, tissue microarray

## Abstract

There is a pressing need for biomarkers for targeted immunotherapy against breast cancer (BCA), the leading cause of cancer death in women. Previously, a blood group precursor O-core epitope gp^Cl^ was found to be highly expressed in breast circulating tumor cells (BCTCs) and BCA cell lines with cancer stem cell (BCSC) features. In this pilot study, the breast tissue distribution of gp^C1^ was examined using tissue microarrays (TMAs). Notably, gp^C1^ positive cells were detected in the major histological types of neoplastic breast tissues. Conversely, none of the breast tissues derived from subjects without BCA were gp^C1^ positive. Thus, gp^C1^ expression seems to be tumor-specific but not histological type-dependent, reflecting perhaps its characteristics as a conserved epitope of oncofetal blood group precursor antigens.

## Introduction

Worldwide, breast cancer (BCA) is the most frequent malignancy and the leading cause of cancer death in women [1, 2]. It is a heterogeneous disease at the molecular level, and the treatment concepts have evolved to include biologically directed therapies [1, 3]. There is an urgent need for biomarkers that can provide information about clinical and pathological features and prognosis to guide treatment stratification [4]. It is well known that abnormal glycosylation is involved in virtually every cancer type [5, 6]. Glycan markers take advantage of surface-exposed and easily accessible cellular features and may be important potential BCA biomarkers in the era of precision therapy. Previously, we investigated the expression of a blood group precursor O-core glyco-epitope gp^C1^ in BCA at the cellular level and demonstrated that gp^C1^ is frequently expressed by a number of human BCA cell lines, as well as by breast circulating tumor cells (BCTCs) in stage IV metastatic BCA patients [5–7]. Interestingly, in a patient with advanced triple-negative BCA, 92.5% of CTCs (37/40 CTCs) were found to be gp^C1^ positive [5].

The high expression rate of gp^C1^ in BCA suggests this blood group precursor antigen may be a BCA biomarker, and the strikingly high percentage of gp^C1^ positive CTCs in the advanced stage of triple-negative BCA indicate gp^C1^ is potentially associated with aggressive tumor behaviour. Hence, in the present study, we further investigated the expression of glyco-epitope gp^C1^ in tissue microarrays from BCA patients who had different histological tumor types classified at various pathological stages.

## Methods and Materials

A key immunological probe of this investigation was the anti-tumor glycan monoclonal antibody (mAb), G1, which opposes epiglycanin, the major sialomucin glycoprotein (~500 kDa) of murine mammary adenocarcinoma TA3 cells and has been shown to specifically bind human carcinoma-associated antigen *in vitro* [8, 9]. As has been seen with mAb AE-3, G1 is highly specific for glyco-epitope gp^C1^ [6]. De-identified human BCA tissue microarrays were obtained from the Cooperative Human Tissue Network (CHTN), mid-Atlantic division (Charlottesville, VA, USA). The immunohistochemistry (IHC) study was performed on formalin-fixed, paraffin-embedded tissue microarray sections using mAb G1. An independent, blinded evaluation was performed by qualified pathologists using three parallel staining results. Statistical analyses were performed using SAS Survey Procedures (SAS 9.4, SAS Institute Inc, Cary, NC, USA). Fisher’s exact test was used for two-group comparisons.

## Results and Discussion

The IHC study was performed on 64 specimens of the tissue microarray; four samples were excluded because there was not enough tissue for interpretation, and eight samples served as controls. A total of 52 breast tissue specimens were included in the analysis (Table 1). As shown in (Table 1), there were 13 non-neoplastic specimens: six were from patients without breast carcinoma (NB-NC), and seven were from patients diagnosed with breast carcinoma (NB-C). No gp^C1^ expression was observed in the NB-NC specimens (as indicated by uniformly negative G1 staining). The tissues from the NB-NC and NB-C group showed a gp^C1^ expression frequency of 2/13 (15.4%), and only one specimen demonstrated focal strong positive staining. The 39 remaining neoplastic specimens exhibited a 26/39 (66.7%) gp^C1^ positive rate, of which 12/39 (30.8%) showed diffuse positive or focal strong positive patterns. Compared to the non-neoplastic specimens, BCA neoplastic specimens had a higher rate of gp^C1^ expression (*P* = 0.0028).

**Figure 1:**
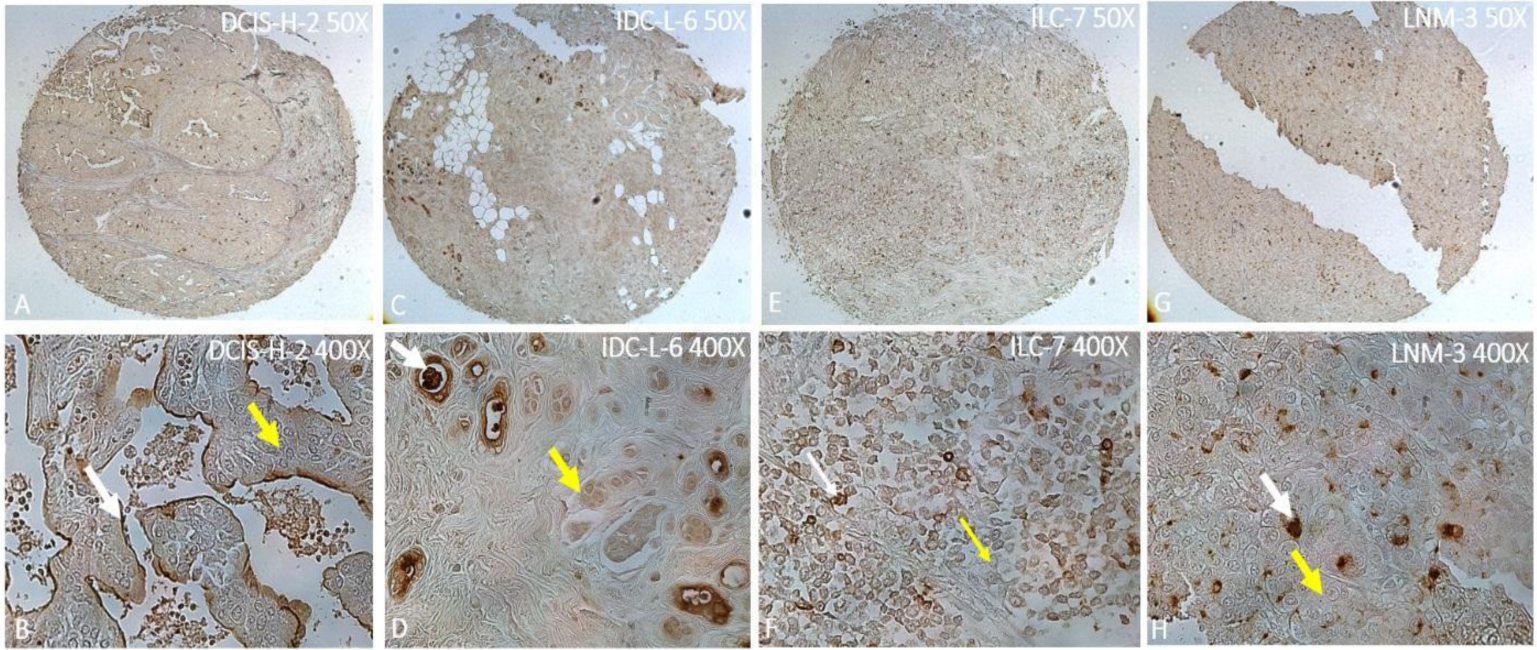
Immunohistochemical study of gp^C1^ expression in primary and metastatic BCA tissue with different pathological grades and histological types by antibody G1. White arrows show positive staining, and yellow arrows show negative staining. **A)** Diffuse positive staining in high-grade DCIS (DCIS-H). **B)** Positive vs. negative staining in an area of DCIS-H. **C)** Diffuse positive staining in low-grade IDC (IDC-L) (grade 1 and 2). **D)** Local strong positive vs. negative staining in an area of IDC-L. **E)** Diffuse positive staining in ILC. **F)** Positive vs. negative staining in an area of ILC. G) Diffuse positive staining in LNM. H) Positive vs. negative staining in an area of LNM.

We examined the pattern of gp^C1^ expression in neoplastic specimens with different pathological grades and histological types. As shown in (Figure 1), gp^C1^ was overexpressed in a subset of BCA cells within each type of positive specimens, while other BCA cells did not stain. Most gp^C1^ positive cells were cytoplasmic or membranous stained. Notably, gp^C1^-positive cancer cells and negative cancer cells co-existed in most of the BCA tissue specimens with the latter as the predominant cell populations while the former appeared like “seeds” in the “lawn” of negatively stained cancer tissues. Clarke *et al.* have demonstrated a cancer stem cell (CSC) model in solid tumors [10]. BCSCs are a small subset of BCA cells and thought to have a central role in the initiation and progression of BCA and in the clinical response to therapy. The BCSC related mutation pathways have been reported to correlate with metastases and the markers CD44/CD24 and ALDH1 have been widely used; however, their expression is not always consistent [11, 12]. The gp^C1^, which is a blood group precursor-based oncofetal antigen, may be explored as a BCSC biomarker candidate, and it showed no cross-reactivity with normal breast tissue in our study [5–7]. In fact, the selective gp^C1^ expression in NB-C specimens may indicate gp^C1^ could serve as a cancer-specific target for use in early detection of BCA even before neoplastic changes.

In conclusion, these findings suggest the gp^C1^ blood group precursor-based oncofetal antigen may be useful in identifying a subset of BCA cells. Further investigation of gp^C1^ expression level, its relationship with BCA progression, and comparisons with traditional BCSC markers are needed. Further exploration of glycan markers for BCTCs/BCSCs is likely to provide new insight into precision medicine and targeted immunotherapy of BCA.

## Figures and Tables

**Table 1:** Results of breast tissue microarray immunohistochemical analysis of gp^C1^ expression.

		gp^C1^ Positive (%)	gp^C1^ Negative (%)	gp^C1^ Diffuse or focal strong positive (%)
Non-neoplastic breast tissue	NB-NC (n = 6)	0(0)	6(100%)	0(0%)
	NB-C (n = 7)	2(28.6%)	5(71.4%)	1(14.3%)
	Non-neoplastic total	2(15.4%)	11(84.6%)	1(7.7%)
Neoplastic breast tissue	DCIS-L (n = 7)	5(71.4%)	2(28.6%)	2(28.6%)
	DCIS-H (n = 7)	4(57.1%)	3(42.9%)	2(28.6%)
	DCIS total	9(64.3%)	5(35.7%)	4(28.6%)
	IDC-L (n = 6)	5(83.3%)	1(16.7%)	3(50%)
	IDC-H (n = 7)	6(85.7%)	1(14.3%)	2(28.6%)
	IDC total	11(84.6%)	2(15.4%)	5(38.5%)
	ILC (n = 5)	3(60%)	2(40%)	2(40%)
	LNM (n = 7)	3(42.9%)	4(57.1%)	1(14.3%)
	Neoplastic total	26(66.7%)	13(33.3%)	12(30.8%)

NB-NC: Non-neoplastic breast tissue from patients without breast carcinoma; NB-C: Non-neoplastic breast tissue from breast carcinoma patients; DCIS-L: Ductal carcinoma *in situ*, low grade; DCIS-H: Ductal carcinoma *in situ*, high grade; IDC-L: Invasive ductal carcinoma, grade 1 or 2; IDC-H: Invasive ductal carcinoma, grade 3; ILC: Invasive lobular carcinoma; LNM: Breast carcinoma metastatic to regional lymph nodes.
